# The Influence of Aeolian Sand on the Anti-Skid Characteristics of Asphalt Pavement

**DOI:** 10.3390/ma14195523

**Published:** 2021-09-24

**Authors:** Jingsheng Pan, Hua Zhao, Yong Wang, Gang Liu

**Affiliations:** 1 College of Water and Architectural Engineering, Shihezi University, Shihezi 832003, China; pjs@stu.shzu.edu.cn (J.P.); jackerry_zhao@163.com (H.Z.); 2 State Key Laboratory of Silicate Materials for Architectures, Wuhan University of Technology, Wuhan 430070, China

**Keywords:** asphalt pavement, sand accumulation conditions, skid resistance, British Pendulum Number (BPN), texture index

## Abstract

The influence of sand accumulation on the skid resistance of asphalt pavement was studied. Many scholars have researched the anti-skid performance of conventional asphalt pavements. However, there is a lack of research on the anti-skid performance of desert roads under the condition of sand accumulation. In this study, AC-13 and AC-16 asphalt mixtures were used. The British Pendulum Number (BPN) under different sand accumulations was measured with a pendulum friction coefficient meter, and the Ames engineering texture scanner was used to obtain different sand accumulations. The texture index of asphalt mixture was used to study the macro and micro texture of asphalt pavement under different amounts of sand accumulation, and the degree of influence of different particle sizes on BPN was obtained through gray correlation analysis. The test results show that the presence of aeolian sand has a significant impact on the macro and micro texture of the asphalt pavement and will cause the anti-skid performance to decrease. Moreover, there is an apparent positive linear correlation between the road surface texture index and BPN. The research results may provide reference and reference for the design and maintenance of desert highways.

## 1. Introduction

The anti-skidding of the pavement is of great significance to traffic safety [1]. The anti-skid performance of the road surface depends on the macro-structure and micro-texture of the road surface, which provides sufficient adhesion for vehicle driving [2,3]. The lithology and angular characteristics of aggregate minerals affect the texture of the road surface [4,5,6]. Specifically, the lithology of the aggregate determines the resistance to abrasion of the road surface under traffic loads, and the angularity affects the micro/macro texture of the road surface. Different rocks have different resistance to abrasion.

The skid resistance of pavement surface is mainly controlled by microstructure and macrostructure, in which microstructure mainly affects the friction at low speed, while macro structure mainly affects the friction at high speed. Many scholars have done relevant research and discussion on the specific quantitative relationship between these two structures and road surface friction coefficient. Friel et al. [7] obtained the surface texture image of coarse aggregate through the scanning electron microscope, analyzed its digital image and fractal dimension by computer, and obtained the influence of micro-texture on pavement skid resistance. Serigost [8] collected many texture data and friction coefficient of Texas highway surface, compared and regressed them, obtained the relationship between pavement friction coefficient and microtexture, and realized the purpose of predicting BPN and friction coefficient with pavement microtexture. Based on the comprehensive analysis of surface characteristics such as friction, texture, and anti-sliding performance of asphalt mixture in laboratory and field, Rezaei [9] proposed a model to characterize anti-sliding performance by mixture gradation, aggregate texture, and traffic volume level. Li [10] studied the changes of aggregate area, volume, and texture parameters of different aggregates before and after microscale wear and polishing with the help of a portable three-dimensional scanner. Ling [11] obtained the pavement surface texture elevation by using the image processing method and analyzed the relationship between different wavelength textures and anti-sliding performance.

In reality, the road surface texture is inevitably affected by external environmental factors, such as rainfall, snowfall, ice, and other pollutants, which affect the friction between the tire and the road [12,13]. In addition, changes in climate and traffic volume significantly impact the texture of the road surface [14,15]. Rain has seriously affected the anti-skid performance of the road surface [16,17,18,19]. The presence of rainwater becomes a lubricant between the tires and the road surface. The membrane generates hydrodynamic pressure on the wheels, which reduces the adhesion of the tire to the road surface. As the thickness of the water film increases, the anti-skid performance decreases more [20]. Under freezing conditions, ice and snow fill the gap on the road surface, reduce the adequate depth of the macrotexture, hinder the direct contact between the tire and the road. The anti-sliding performance is seriously reduced [21,22,23,24], resulting in the loss of the original stability of vehicle braking and the increase of braking distance, which brings hidden dangers for driving safety. Other pollutants such as sand, salt, and oil leakage will also affect the anti-skid performance of the road [25,26]. It blocks the gaps of the asphalt pavement, reduces the texture of the road surface, and makes the pavement lose its original anti-skid ability. In addition, the change of temperature will also affect the anti-sliding performance of pavement [27,28]. In the case of clean road surface, the anti-skid performance in summer is generally lower than in winter. The above are the problems faced by the anti-skid performance of asphalt pavement in conventional areas. The anti-skid performance of the pavement is easily disturbed by the external environment.

Xinjiang is a typical arid and semi-arid area in China, with severe desertification. In the desert area, the harm of wind sand to roads is particularly prominent [29]. Among them, aeolian sand covering the road surface is one of the typical hazards. The research shows that the contact area will affect the anti-sliding performance in a particular proportion [30]. Aeolian sand reduces the effective contact area between the tire and the road surface, in which the larger particles cover the road surface and hinder the direct contact between the tire and the road surface, and the smaller particles fill the macrostructure of the road surface and reduce the friction coefficient. As a result, the skid resistance of asphalt pavement is seriously reduced, and sand accumulation has become an important reason for weakening the function of the road surface.

Desert roads are a unique form of the road in extreme climate environments, which accounts for a relatively small number globally, but the harm of wind sand to the highway can not be ignored. Compared with the research on the anti-sliding performance of conventional asphalt pavement, the literature on the anti-sliding performance of desert highways under sand accumulation is more minor. This study took the sand accumulation into account on desert highways to analyze the morphological characteristics of aeolian sand. The texture change trend of asphalt pavements and the evolution of anti-skid performance were studied under different amounts of sand accumulation. Meanwhile, the impact of aeolian sand granularity was considered. The research results are expected to provide a reference for the prevention and control of desert highway diseases, especially the anti-skid performance.

## 2. Materials and Methods

### 2.1. Materials

Based on the Test Methods of Aggregate for Highway Engineering [31], the properties of aggregate were tested, and their results are given in Table 1. The aggregate used in this study is a conglomerate from Xinjiang of China.

**Figure 1:**
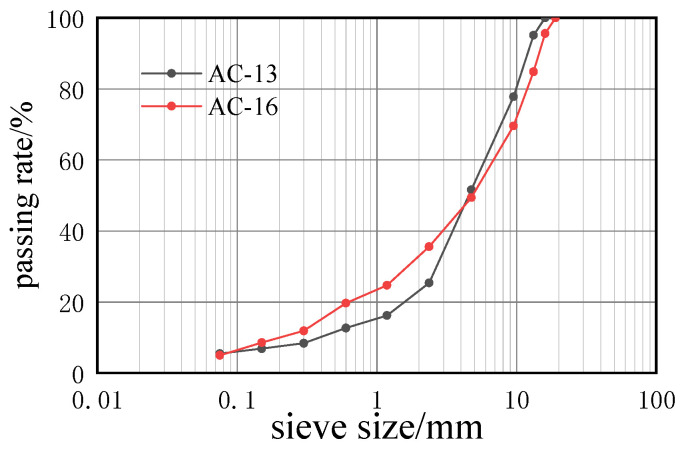
Aggregate gradation.

In this paper, two types of asphalt mixtures were prepared, AC-13 and AC-16. The aggregates are both conglomerates and have the same specific gravity. The passing rate of each sieve is shown in Figure 1. The optimal binder content of AC-13 is 4.7%, the air voids content 4.3%, the optimal binder content of AC-16 4.2%, and the air voids content is 4.3%.

**Figure 2:**
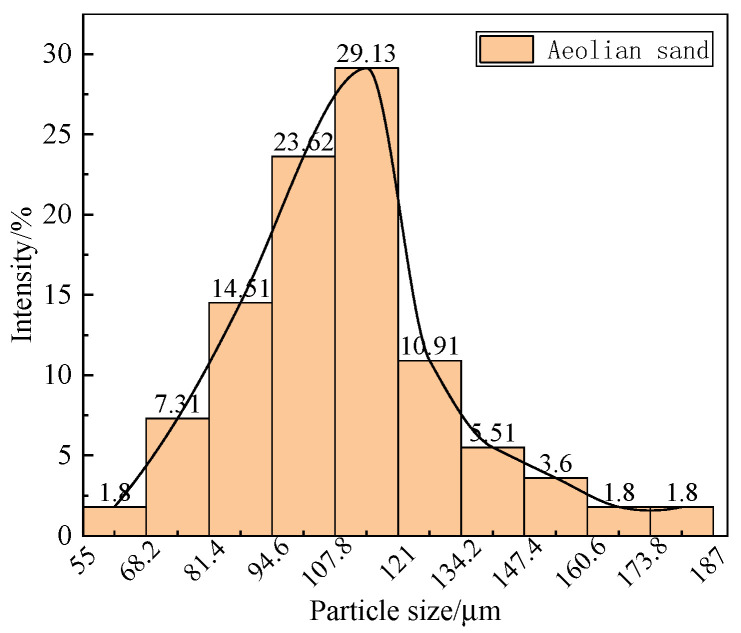
Particle size distribution of aeolian sand.

**Figure 3:**
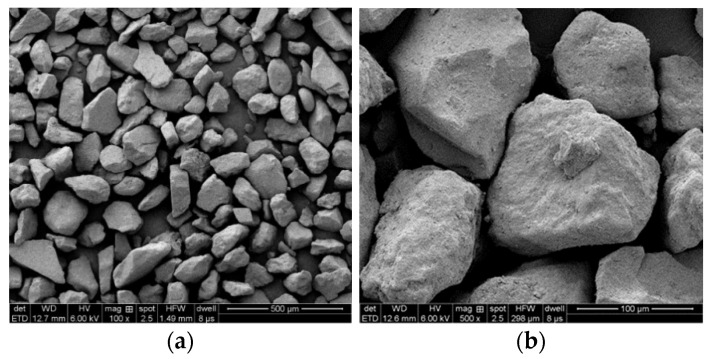
SEM images of aeolian sand. (**a**) SEM image of 100 times; (**b**) SEM image of 500 times.

The sand sample comes from aeolian sand-covered on the road surface in the desert area of Xinjiang. In order to explore the different particle size content and microscopic morphology of aeolian sand, the particle size analysis of aeolian sand was carried out, and the particle size distribution of the sample was tested with a laser particle size analyzer (instrument model: Master-sizer 2000) (Malvern Instruments Ltd., Worcestershire, UK). The results are shown in Figure 2 by using the field emission scanning electron microscope (Quanta FEG 250) (FEI Co., Hillsboro, OR, USA). The morphology of aeolian sand under different magnifications is shown in Figure 3.

According to the results obtained by the laser particle size analyzer, the maximum particle size of aeolian sand is 187 μm, and the minimum is 55 μm. Among them, the particle size distribution of aeolian sand between 94.6–121 μm accounts for the most significant proportion, which is 52.8%. From Figure 3, it can be observed that the aeolian sand particles are relatively granular, which can provide a rolling medium for the rolling friction between the wheel and the road surface and reduce the anti-skid performance of the road surface when it exists on the road surface.

### 2.2. Test Method

The pendulum friction coefficient instrument was used to measure the British Pendulum Number (BPN) of the surface of the laboratory compacted slab (300 mm × 300 mm × 50 mm) intended for wheel track rutting test with different sand accumulation [32].

**Figure 4:**
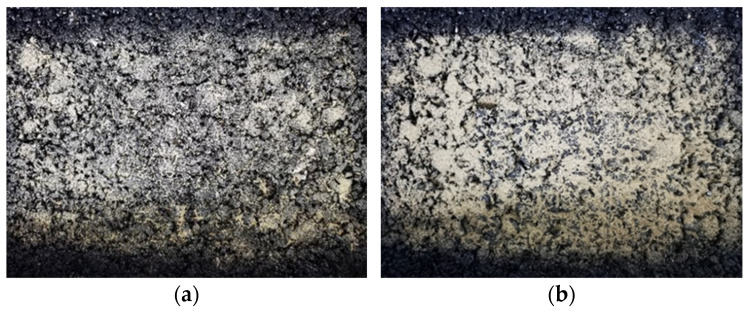
The accumulation patterns of different aeolian sands are 30 g (**a**) and 70 g (**b**), respectively.

In order to study the impact of sand accumulation on the anti-skid performance of the pavement, a rut plate specimen was made indoors, and the sand on the road area was simulated through different amounts of sand, and the BPN value under different conditions was tested, as shown in Figure 4. Moreover, the different amounts of sand accumulation were 0 g, 10 g, 20 g, 30 g, 40 g, 50 g, 60 g, 70 g, and 80 g, respectively. Before the test, clean the surface of the test piece with a brush and then spread the sand weighed in advance evenly over the whole test piece. After the test, clean the sand on the surface of the test piece with a brush and repeat this method many times until the test was completed.

**Figure 5:**
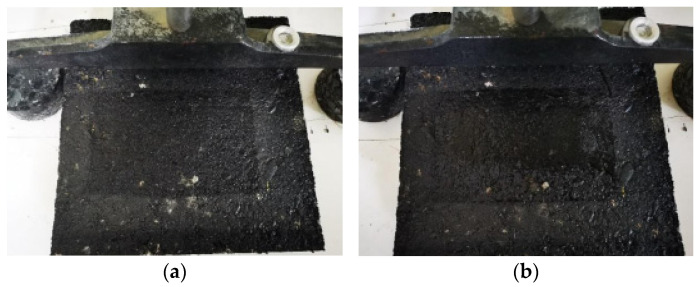
Specimens with water content of 5% (**a**) and 10% (**b**) respectively before adding aeolian sand.

**Figure 6:**
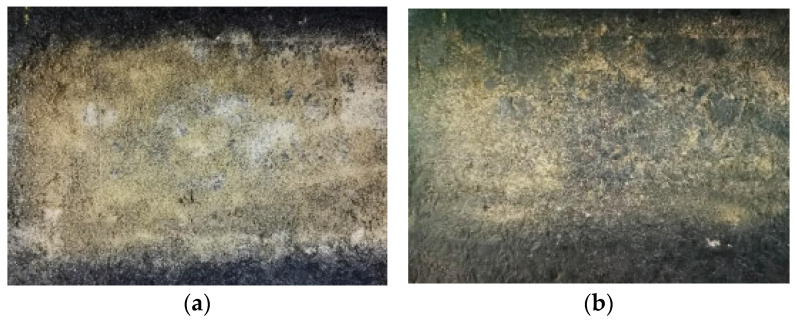
Specimens with a water content of 5% (**a**) and 10% (**b**) respectively after adding aeolian sand.

As shown in Figure 5 and Figure 6, we prepared aeolian sand samples with moisture contents of 5% and 10%, respectively, and used the pendulum friction coefficient meter to determine the BPN value under different sand accumulation in order to study the influence of the moisture content of aeolian sand on the anti-sliding performance.

**Figure 7:**
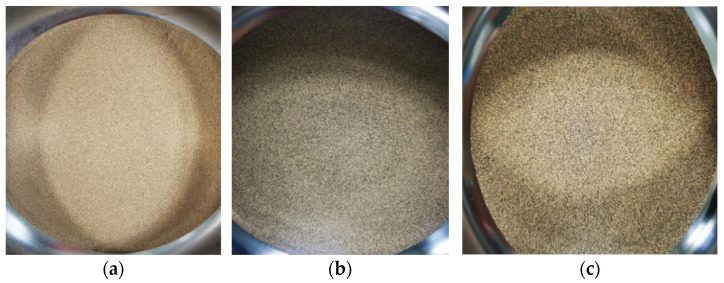
Aeolian sand with different particle size ranges less than 0.075 mm (**a**), 0.075–0.15 mm (**b**), and greater than 0.15 mm (**c**), respectively.

In order to study the influence of different particle diameters of aeolian sand on the anti-skid performance of two types of asphalt mixtures, the aeolian sand samples were first screened. The particle size distribution of aeolian sand was mainly divided into three ranges, namely less than 0.075 mm, 0.075–0.15 mm, and greater than 0.15 mm, as shown in Figure 7. The mass percentages are 12.5%, 83.6%, and 3.9%, respectively. Then, the BPN values of the three particle sizes of aeolian sand were tested separately on the road surface.

**Figure 8:**
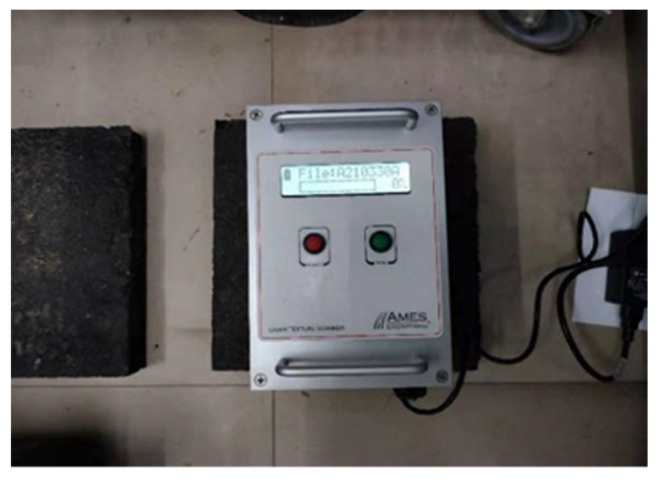
Ames texture scanner.

The Ames engineering texture scanner (LTS 9400) (Ames Engineering Co., Ames, IA, USA) was used to detect the texture indicators of the specimens under different amounts of sand. As shown in Figure 8, select 30 scan lines among them, and select the three texture indicators of MPD, Ra, and Rq. Among them, MPD is the mean profile depth of the specimen surface, Ra is the average deviation of the contour arithmetic, and Rq is the root mean square deviation of the profile.

## 3. Results and Discussion

### 3.1. BPN Test

**Figure 9:**
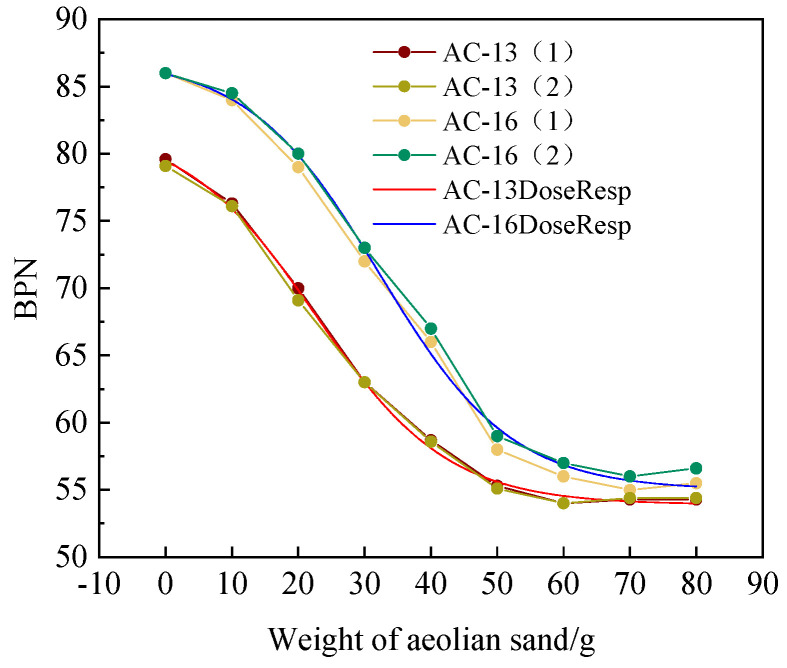
BPN value under different aeolian sand weight.

As shown in Figure 9, the BPN values of the two types of specimens change under different amounts of sand. The amount of sand is inversely proportional to the BPN value on the surface of the specimen. The BPN value decreases with the increase in the amount of sand. The BPN values of the two asphalt mixture types show the same change law, which can be divided into three stages. In increasing the initial sand volume from 0 to 10 g, the BPN value decreased slowly. The BPN value of AC-13 decreased by 4.1% and 3.8%, and the BPN value of AC-16 decreased by 2.3% and 1.7%, respectively. Then, when the sediment volume increased from 10 g to 50 g, the decreasing range of BPN value of the two mixtures increased obviously, in which the BPN value of AC-13 decreased by 27.5% and 27.6%, and the BPN value of AC-16 decreased by 31% and 30.2%. In the later stage, when the amount of sedimentation reached 60 g, the BPN value decreased slowly, and the final change curve gradually became flat. The BPN value of the two mixtures showed a slight rebound, of which AC-13 rebounded by 0.6% and 0.7%, and AC-16 rebounded 0.9% and 1.1%.

The DoseResp model is used to fit the BPN value change curves of the two mixtures. The model equations and various parameters are shown in Table 2. The goodness of fit of the two mixtures is above 0.99, indicating that the changes in the BPN value of the two mixtures under different amounts of sedimentation conform to the model. The model can predict the trend of the BPN value of AC-13 and AC-16 well under the conditions of sedimentation.

The analysis believes that due to the generally small particle size of aeolian sand, it enters the gaps of the asphalt mixture at the initial stage and blocks the gaps, which reduces the depth of the structure and decreases the anti-skid performance. Then, as the quality of the aeolian sand increases, the gap is gradually filled, and part of the aeolian sand covers the road surface. Due to its round appearance, the tire and the road surface form a micro-bearing system, which changes sliding friction into rolling friction, and the friction force drops sharply. The main factor causing the decrease of friction is that the aeolian sand is not adhered to the adhesive asphalt, resulting in rolling friction under the impact force of the pendulum. Finally, when the amount of accumulated sand increases to a certain level (60 g), the friction is controlled by the accumulated sand. The accumulated sand produces resistance to the rubber slider of the pendulum friction coefficient meter, causing its BPN value to rise. The reason for this phenomenon may be the limitation of the test equipment, which does not conform to the actual situation.

**Figure 10:**
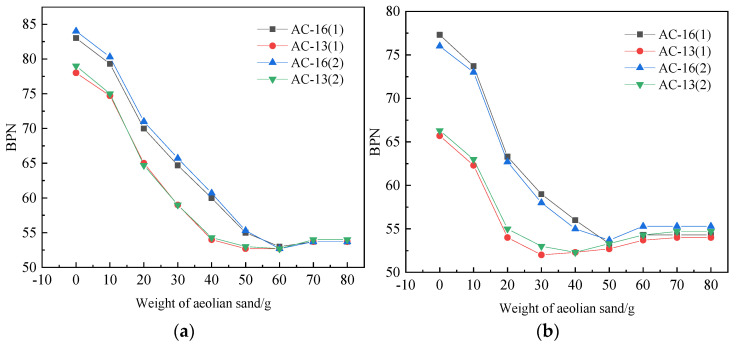
BPN under different sediment accumulation conditions in the wet state. (**a**) The moisture content of accumulated sand is 5%; (**b**) The moisture content of accumulated sand is 10%.

As shown in Figure 10a, when the amount of aeolian sand with the water content of 5% increases to 10 g, the BPN values of the two mixtures decrease by 4.9% and 4.5%, respectively. At this time, the AC-13 decreases. The amplitude is more significant than AC-16. The analysis believes that the air voids content and texture depth of AC-13 is less than AC-16. Under the interference of water and sand, the decrease of BPN value is slightly more significant than AC-16. With the increase of sand accumulation, the anti-sliding performance of the two mixtures decreases and finally tends to be stable. During the whole process, the BPN values of the two mixtures decrease by 28.5% and 35.7%, respectively. When the water content of aeolian sand is 10%, as shown in Figure 10b, the initial BPN value of the two mixtures decreases overall. The initial value of AC-13 is significantly lower than AC-16. When the amount of sand increased to 30 g, the BPN value droped to the lowest point. The BPN value of AC-16 reached the lowest point when the amount of sand increased to 50 g. There was a slight rebound. Among them, the BPN value of AC-13 rebounded by 3.1% and 4.4%, and the BPN value of AC-16 rebounded. The margins were 1.9% and 2.4%. The analysis shows that water becomes the lubricant between the sand, which makes the micro-bearing system formed by the wheel sand road more significant. The wet sand exists between the wheel and the road like a ball, and the anti-sliding ability drops sharply at this time.

Compared with the dry aeolian sand acting on the road surface, the aeolian sand in the wet state makes the BPN value of the two mixtures generally lower. At this time, the pavement voids and macrostructures are not only filled with sand but filled with sand and water. This process accelerates the decrease in the depth of the pavement structure, allowing more sand and water to act on the road surface and hinder the tires and the pavement. The direct contact between the sand and the moisture exists in the sand surface and the gaps, which provides sufficient lubrication between the sand and between the sand and the wheel and the road surface, so the BPN value can quickly drop to the lowest point.

**Figure 11:**
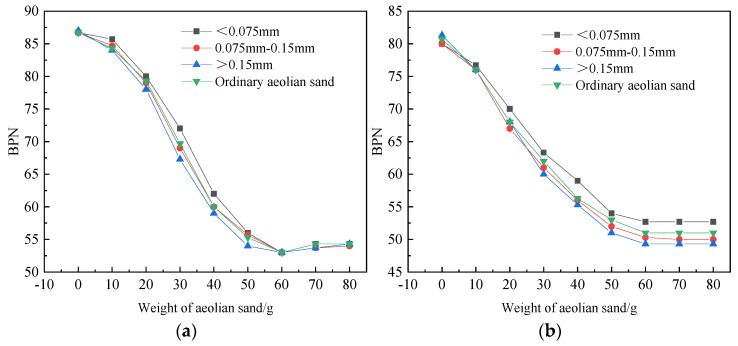
BPN value of aeolian sand with three particle sizes existing alone on the road surface. (**a**) BPN value of aeolian sand with three particle sizes existing alone on the AC-16 pavement surface; (**b**) BPN value of aeolian sand with three particle sizes existing alone on the AC-13 pavement surface.

Figure 11a shows the variation law of BPN values of three particle sizes of AC-16 under different sediment volumes. On the whole, the variation trend of three particle sizes is the same as that of conventional aeolian sand samples. However, the slope of the BPN value curve for aeolian sand less than 0.075 mm in the initial stage is the smallest, while the curve greater than 0.15 mm has the most significant variation range, and the curve decreases rapidly in the whole process. In the end, there was a significant recovery. In increasing the amount of sand from 0 to 10 g, the BPN corresponding to the three particle sizes decreased by 4.1%, 5.0%, and 6.5%, respectively. When the amount of sand increased from 10 g to 50 g, the three particle sizes, the corresponding BPN dropped by 29.6%, 31.6%, and 30.2%, respectively. The overall decline in Figure 11b is smaller than that in Figure 11a, indicating that AC-16 is more disturbed by wind and sand. However, the slope of the BPN curve at the initial stage was less than that of AC-13, and three particle sizes decreased by 1.2%, 2.3%, and 3.4%, respectively. The analysis shows that the macro structure of AC-16 is larger than AC-13, and it can contain aeolian sand particles. Relatively strong, the decrease in BPN value is slight in the initial stage. Then, when the macro structure was filled with aeolian sand particles, the BPN value dropped sharply, and the three particle sizes dropped by 34.7%, 34.2%, and 35.7%, respectively. When the BPN value of the two mixtures fell to the lowest point in the later period, because the surface texture of AC-16 was slightly larger than AC-13, the BPN value of AC-16 rose slightly.

The decrease of the BPN value on the surface of the test piece is related to the particle size. The larger the particle size, the more significant the decrease of the BPN value on the surface of the test piece. The results indicated that the larger the particle size of the aeolian sand, the greater the ability to fill the gap and the surface structure of the specimen. It reduced the macrostructure of the specimen surface and its anti-sliding ability. The BPN value decreases more rapidly. Therefore, under the same filling rate, the larger the particle size of the aeolian sand, the faster the anti-skid performance of the pavement decreases.

**Figure 12:**
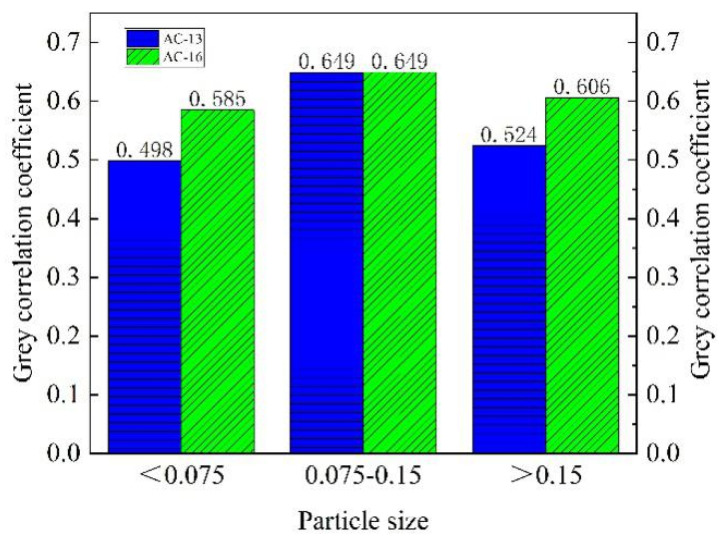
Grey correlation analysis results.

In order to determine the degree of influence of the three particle sizes on the BPN value, the test results were analyzed with a gray correlation. The BPN value under the three particle sizes was used as the comparison sequence. The BPN value under the conventional aeolian sand was used as the reference sequence for comparative analysis. The results are shown in Figure 12.

It can be seen from Figure 12 that the gray correlation coefficient of particles less than 0.075 mm in the two kinds of asphalt mixture is the smallest, which means that the particle size has the least influence on the BPN value. In the conventional aeolian sand, the particle size less than 0.075 mm accounts for a relatively small proportion. In addition, its particle size is small, which mainly fills the gaps in the road surface and contributes little to the rolling medium between the tire and the road surface. The 0.075–0.15 mm particles have an essential impact on the BPN value. They had a large mass proportion, and acted as a rolling medium for the rolling friction between the tire and the road surface. Although the particles larger than 0.15 mm had the smallest proportion, they played a significant supporting role between the wheel and the road surface, disturbing the BPN value. The gray correlation coefficient corresponding to the particle larger than 0.15 mm was in the middle level.

### 3.2. Texture Index

The most intuitive impact of aeolian sand on the pavement is to change its original macroscopic structure. Meanwhile, smaller particles enter the pavement voids to fill the macroscopic structure and reduce the structure depth, thereby reducing the friction coefficient. On the other hand, larger particles with diameters covering the road surface hinder the direct contact between the tire and the road surface and reduce the effective contact area between the tire and the road surface, thereby reducing the anti-skid performance.

**Figure 13:**
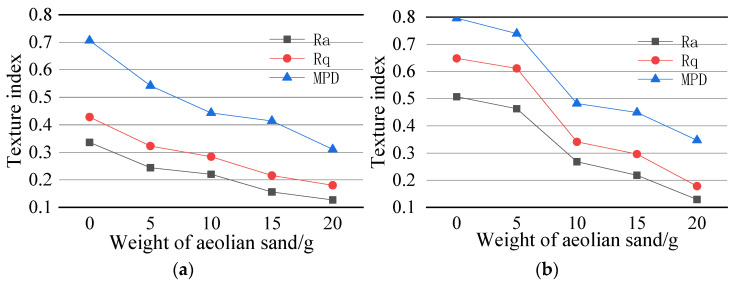
Texture index under different sand accumulation. (**a**) Texture index under different sand accumulation on the AC-13 pavement surface; (**b**) Texture index under different sand accumulation on the AC-16 pavement surface.

In order to further understand the road texture under different sand accumulation, the Ames engineering texture scanner (LTS 9400) was used to scan the surface of specimens under different sand accumulation. Thirty scanning lines were selected for each specimen to extract three texture indexes, namely MPD, Ra, and Rq. Figure 13a,b are the change trends of texture indicators of AC-13 and AC-16 mixtures under different sand accumulations. The sizes of the three indicators are inversely proportional to the sand accumulation, and Ra, Rq. The similarity of the change curves of the two indicators is relatively high, indicating that the two are affected by the same degree of sand accumulation.

MPD reflects the average section depth of the specimen surface. With the increasing amount of aeolian sand, the texture depth of the specimen surface is gradually reduced, and the surface texture changes passively. At this time, MPD decreases with the trend, and the BPN value greatly correlates with the surface texture of the specimen, which also decreases. The MPD of AC-13 in Figure 13a and AC-16 in Figure 13b decreased by 23% and 7.2%, respectively, when the initial sand accumulation increased from 0 g to 5 g. Then, with the gradual increase of the amount of aeolian sand on the specimen surface, the MPD decreased. During the whole process, the MPD of the two specimens decreased by 52% and 56.4%, respectively.

Ra is the average deviation of the contour arithmetic and is used to characterize the texture of the road surface in the scanning range. It reflects the dispersion degree of the change of the pavement structure profile concerning the reference line. The increase of aeolian sand makes the surface part of the specimen to be filled. As the depth decreases, the texture decreases. When aeolian sand increased from 0 g to 5 g, the Ra of the two mixtures decreased by 27.4% and 8.6%, respectively. During the whole process, the Ra of the two mixtures decreased by 62.2% and 74.6%, respectively. As shown in Figure 13, the Ra value of AC-16 is more significant than that of AC-13, and it sharply drops with the increase of sediment accumulation.

Rq is the root mean square deviation of the profile and is used to indicate the surface texture of the specimen. As the amount of aeolian sand gradually increases, the internal voids of the specimen are occupied by aeolian sand particles, the macrostructure is reduced, and the surface is rough. When the temperature decreases, the Rq index decreases, and then the BPN value of the specimen surface decreases. In the initial stage, Rq of AC-16 decreased slowly, compared with AC-13. The decrease of Rq index was 24.5% and 5.7%, respectively, and the decline of AC-16 in the late stage was significantly increased, and the overall decline was more than AC-13. During the whole process, the Rq of AC-13 and AC-16 decreased by 57.9% and 72.5%, respectively.

On the whole, when the number of sand increases from 0 g to 5 g, the three texture indicators of MPD, Ra, and Rq decrease by AC-13 larger than AC-16. Meanwhile, the voids and macro-structure of AC-13 are smaller than AC-16. In the presence of a small amount of aeolian sand, the texture index of the AC-13 surface is more sensitive. AC-16 can contain a small amount of sand and has little effect on its texture in the initial stage.

Aeolian sand covers the road surface, which directly affects the macroscopic structure of the road surface, which in turn makes the road surface texture change significantly. The result of the change in the road sign texture is the corresponding decrease in the BPN value. Therefore, it is believed that the relationship between the road surface texture index and anti-skid performance certainly exist.

**Figure 14:**
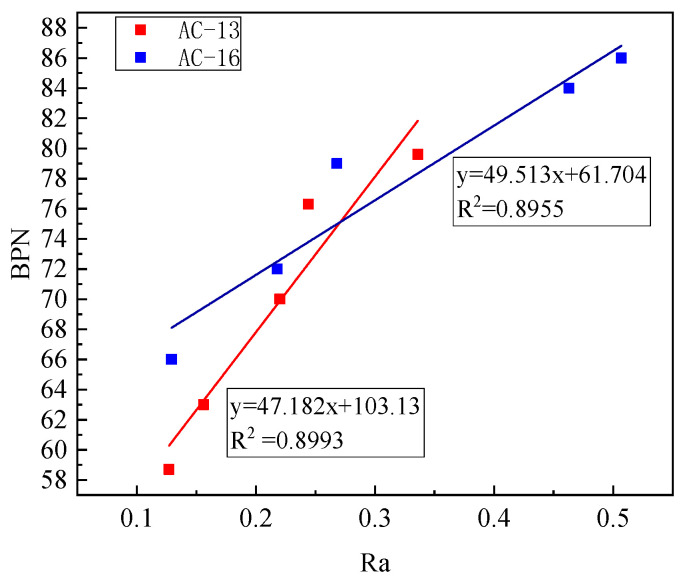
Relationship between BPN and Ra.

The correlation analysis between the BPN value and three texture indicators of MPD, Ra, and Rq was performed. As shown in Figure 14, BPN and Ra values have an excellent linear relationship, indicating that the increase of aeolian sand make the contour of the pavement structure. The slope of the trend line for AC-16 is more significant than that for AC-13.

**Figure 15:**
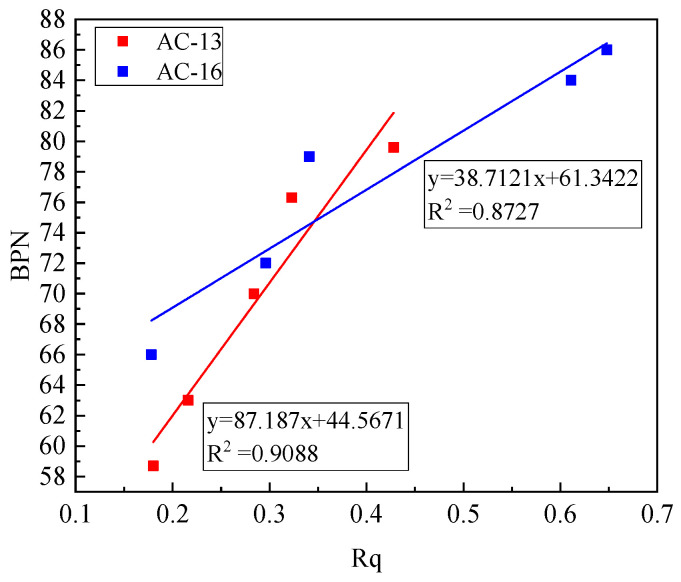
Relationship between BPN and Rq.

The BPN value and Rq of the two mixtures were fitted, and the results are shown in Figure 15. There was also an excellent linear relationship between the BPN value and Rq. The goodness of fit for AC-13 is 0.9088, and for AC-16, 0.8727. The increase of the contoured root means square deviation Rq will cause the increase of the BPN value. The two are in direct proportion, indicating that the more the amount of aeolian sand on the road surface, the stronger the filling capacity of the road surface. At this time, the road surface texture decreases, and the BPN value also increases. It decreases accordingly.

**Figure 16:**
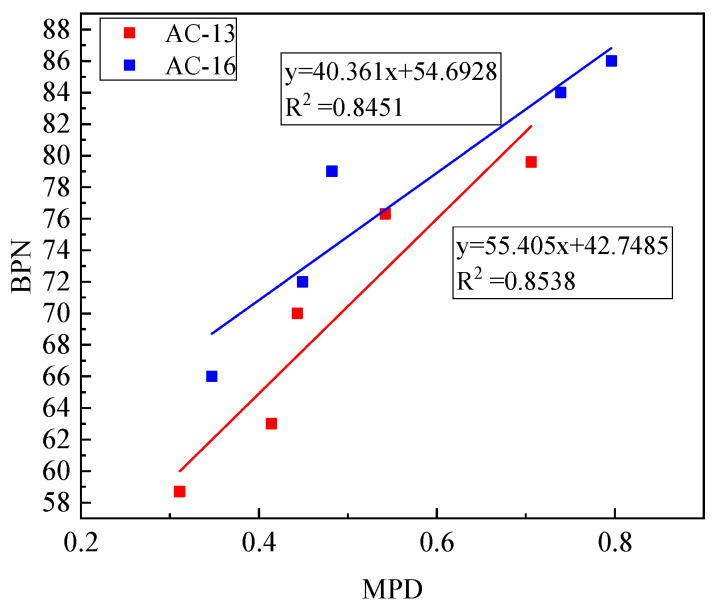
Relationship between BPN and MPD.

Figure 16 shows the results of fitting the BPN value and MPD of the two mixtures. The results show that the BPN value and MPD also have a good linear relationship. The goodness of fit of the two mixtures is above 0.84. The average section depth of MPD decreases with the decrease of the BPN value. The analysis shows that as the number of aeolian sand increases, the texture depth of the specimen surface gradually decreases, and the surface texture changes passively. At this time, the MPD decreases accordingly.

## 4. Conclusions

This paper studies the influence of aeolian sand on the anti-sliding performance and texture index of asphalt mixture, which is a study based on the macro perspective of the anti-sliding performance changes caused by different amounts of sand and asphalt mixture types.

- As the number of sand accumulation increases, the BPN values of AC-13 and AC-16 mixtures show a downward trend. By fitting the DoseResp model, the later changes can be predicted based on the changes in the initial BPN value.
- By studying the anti-sliding performance of pavement surface whether aeolian sand contains water, we find that the sand with water is generally lower than that of dry sand, and the skid resistance decreases with the increase of water volume.
- Through the grey correlation analysis, it is found that the aeolian sand particles between 0.075–0.15 mm have the most significant influence on the BPN value of the two mixtures because its mass percentage is the largest.
- Ames engineering texture scanner was used to obtain texture indicators under different amounts of sand. It is found that all three texture indicators are correlated with the BPN value. With the increase of the sand deposition within 20 g, the effective structural depth of pavement macrotexture decreases, resulting in the decrease of BPN value of road surface, which means that a certain amount of aeolian sand has a significant impact on the anti-skid performance of the mixture at the macro level.

## Figures and Tables

**Table 1:** Aggregate properties.

Test Items	Results	Technical Requirements
Crushed value (%)	12.8	≤26
Weared value (%)	10.5	≤28
Polished value (PSV)	38	≥36
Angularity (s)	31.8	≥30

**Table 2:** Fitting curve of BPN value change under different aeolian sand weight.

Mixture Type	Fitting Equation	Fitting Precision
AC-13	$y=53.911\;+\;\frac{28.182}{{1\;+\;10}^{0.044x\;-\;0.996}}$	0.998
AC-16	$y=54.994\;+\;\frac{32.141}{{1\;+\;10}^{0.044x\;-\;1.420}}$	0.995

## Data Availability

No new data were created or analyzed in this study, data sharing is not applicable to this article.

## References

[1] Anupam K., Tang T., Kasbergen C., Scarpas A., Erkens S. (2020). 3-D Thermomechanical Tire–Pavement Interaction Model for Evaluation of Pavement Skid Resistance. Transp. Res. Rec..

[2] Chu L.J., Fwa T.F. (2016). Pavement skid resistance consideration in rain-related wet-weather speed limits determination. Road Mater. Pavement Des..

[3] Dan H.-C., He L.-H., Xu B. (2015). Experimental investigation on skid resistance of asphalt pavement under various slippery conditions. Int. J. Pavement Eng..

[4] Do M., Cerezo V., Zahouani H. (2014). Laboratory test to evaluate the effect of contaminants on road skid resistance. Proc. Inst. Mech. Eng. Part J.

[5] Eisenberg D. (2004). The mixed effects of precipitation on traffic crashes. Accid. Anal. Prev..

[6] Geedipally S.R., Das S., Pratt M.P., Lord D. (2020). Determining Skid Resistance Needs on Horizontal Curves for Different Levels of Precipitation. Transp. Res. Rec..

[7] Friel J.J., Pande C.S. (1993). A direct determination of fractal dimension of fracture surfaces using scanning electron microscopy and stereoscopy. J. Mater. Res..

[8] Serigos P.A., de Fortier Smit A., Prozzi J.A. (2014). Incorporating surface microtexture in the prediction of skid resistance of flexible pavements. Transp. Res. Rec..

[9] Rezaei A., Masad E. (2013). Experimental-based model for predicting the skid resistance of asphalt pavements. Int. J. Pavement Eng..

[10] Li Q.J., Zhan Y., Yang G., Pittenger D.M., Wang K.C.P. (2019). 3D Characterization of Aggregates for Pavement Skid Resistance. J. Transp. Eng. Part B Pavements.

[11] Chen D., Ling C., Wang T., Su Q., Ye A. (2018). Prediction of tire-pavement noise of porous asphalt mixture based on mixture surface texture level and distributions. Constr. Build. Mater..

[12] Gierasimiuk P., Wasilewska M., Gardziejczyk W. (2021). A Comparative Study on Skid Resistance of Concrete Pavements Differing in Texturing Technique. Materials.

[13] Mataei B., Zakeri H., Zahedi M., Nejad F.M. (2016). Pavement Friction and Skid Resistance Measurement Methods: A Literature Review. Open J. Civ. Eng..

[14] Kienle R., Ressel W., Götz T., Weise M. (2018). The influence of road surface texture on the skid resistance under wet conditions. Proc. Inst. Mech. Eng. Part J..

[15] Kogbara R.B., Masad E.A., Kassem E., Scarpas A. (2018). Skid Resistance Characteristics of Asphalt Pavements in Hot Climates. J. Transp. Eng. Part B Pavements.

[16] Li C., Wang Y., Lei J., Xu X., Wang S., Fan J., Fan S. (2020). Damage by wind-blown sand and its control measures along the Taklimakan Desert Highway in China. J. Arid. Land.

[17] Li P., Yi K., Yu H., Xiong J., Xu R. (2021). Effect of Aggregate Properties on Long-Term Skid Resistance of Asphalt Mixture. J. Mater. Civ. Eng..

[18] Lubis A.S., A Muis Z., Gultom E.M. (2018). The effect of contaminant on skid resistance of pavement surface. IOP Conf. Ser. Earth Environ. Sci..

[19] Nicolosi V., D’Apuzzo M., Evangelisti A. (2020). Cumulated frictional dissipated energy and pavement skid deterioration: Evaluation and correlation. Constr. Build. Mater..

[20] Pomoni M., Plati C., Loizos A., Yannis G. (2020). Investigation of pavement skid resistance and macrotexture on a long-term basis. Int. J. Pavement Eng..

[21] Iii H.R., Nassiri S., AlShareedah O., Yekkalar M., Haselbach L. (2019). Evaluation of skid resistance of pervious concrete slabs under various winter conditions for driver and pedestrian users. Road Mater. Pavement Des..

[22] Tang T., Anupam K., Kasbergen C., Scarpas A., Erkens S. (2019). A finite element study of rain intensity on skid resistance for permeable asphalt concrete mixes. Constr. Build. Mater..

[23] Wang D., Zhang Z., Kollmann J., Oeser M. (2018). Development of aggregate micro-texture during polishing and correlation with skid resistance. Int. J. Pavement Eng..

[24] Wang H., Wang C., Bu Y., You Z., Yang X., Oeser M. (2020). Correlate aggregate angularity characteristics to the skid resistance of asphalt pavement based on image analysis technology. Constr. Build. Mater..

[25] Wang Y., Liu Y., Cheng Y., Xue J. (2020). Evaluation of the Decay Characteristics of Pavement Skid Resistance Using Three-Dimensional Texture from Accelerated Abrasion Test. J. Transp. Eng. Part B Pavements.

[26] Wu J., Wang X., Wang L., Zhang L., Xiao Q., Yang H. (2020). Temperature Correction and Analysis of Pavement Skid Resistance Performance Based on RIOHTrack Full-Scale Track. Coatings.

[27] Yan B., Mao H., Zhong S., Zhang P., Zhang X. (2019). Experimental Study on Wet Skid Resistance of Asphalt Pavements in Icy Conditions. Materials.

[28] Yu M., You Z., Wu G., Kong L., Liu C., Gao J. (2020). Measurement and modeling of skid resistance of asphalt pavement: A review. Constr. Build. Mater..

[29] Yun D., Hu L., Tang C. (2020). Tire-Road Contact Area on Asphalt Concrete Pavement and Its Relationship with the Skid Resistance. Materials.

[30] Zhu X., Yang Y., Zhao H., Jelagin D., Chen F., Gilabert F.A., Guarin A. (2020). Effects of surface texture deterioration and wet surface conditions on asphalt runway skid resistance. Tribol. Int..

[31] Ministry of Transport of the People’s Republic of China (2005). Test. Methods of Aggregate for Highway Engineering (JTG E42-2005).

[32] Ministry of Transport of the People’s Republic of China (2011). Standard Test Methods of Bitumen and Bituminous Mixtures for Highway Engineering (JTG E20-2011).

